# Injury in Children with Developmental Disorders: A 1:1 Nested Case–Control Study Using Multiple Datasets in Taiwan

**DOI:** 10.3390/ijerph19169814

**Published:** 2022-08-09

**Authors:** Shang-Ku Chen, Li-Min Hsu, Nan-Chang Chiu, Wafaa Saleh, Chih-Wei Pai, Ping-Ling Chen

**Affiliations:** 1 Graduate Institute of Injury Prevention and Control, College of Public Health, Taipei Medical University, Taipei 110, Taiwan; 2 Department of Surgery and Traumatology, National Taiwan University Hospital, Taipei 100, Taiwan; 3 Department of Pediatrics, MacKay Memorial Hospital, Taipei 104, Taiwan; 4 Transport Research Institute, Edinburgh Napier University, Edinburgh EH11 4DY, UK

**Keywords:** child injury, developmental disorder, mental illness, drug use

## Abstract

Although past studies have identified predictors related to child injuries with developmental disorders, national-level research in Asia is limited. The objective of this study was to explore the risk factors for child injuries with developmental disorders in Taiwan using a national-level integrated database for the period between 2004–2015 (The Maternal and Child Health Database, National Health Insurance Research Database, Census Registry, and Indigenous Household Registration). Children younger than 12 years old who had records of visiting the ER or being hospitalized due to injury or without injury were included in this study. A 1:1 nested case-control study (injury vs. noninjury) to examine the risk factors for child injury with developmental disorder was performed. A total of 2,167,930 children were enrolled. The risk factors were associated with repeated ER visits or hospitalization: being indigenous (adjusted odds ratio [AOR]: 1.51; CI: 1.45–1.57); having a developmental disorder (AOR: 1.74; CI: 1.70–1.78); and having parents with illicit drug use (AOR: 1.48; CI: 1.32–1.66), alcohol abuse (AOR: 1.21; CI: 1.07–1.37), or a history of mental illness (AOR: 1.43; CI: 1.41–1.46). Being indigenous, having developmental disorders, and having parents with history of illicit drug use, alcohol abuse, or mental illness were predictors related to injuries in children.

## 1. Introduction

Injury is a leading cause of disease burden among children and adolescents worldwide [1]. The global estimate of over 522,000 (80.5/100,000) children under 5 years who died due to injuries in 2015 is higher than previously reported [2]. Unintentional injuries, defined as injuries that are not deliberately inflicted, account for almost 90% of these cases. The causes of injuries include falls, accidental ingestion of poison, drowning, burns, and traffic accidents. In 2013, unintentional injuries accounted for 15.4% of approximately 2.6 million deaths recorded for children aged 1 to 14 years [3]. Moreover, unintentional injury is the leading cause of deaths in all age groups from 1 to 44 years [1].

Child developmental disorders include attention-deficit/hyperactivity disorder (ADHD), epilepsy, autism spectrum disorder, Tourette’s syndrome, and other developmental delay disorders. The prevalence of child developmental disorders in Taiwan and the USA were 11.3% and 16.5%, respectively [4,5]. Children with disabilities have a higher risk of injury than those without disabilities [6,7,8]. In particular, children with emotional or behavioral problems have elevated injury risks. Furthermore, children with developmental disorders such as attention-deficit/hyperactivity disorder (ADHD), epilepsy [9], autism [10], psychopathology [11,12], and perceptual and communication impairments, such as hearing and visual limitations [8,13,14], also have increased injury rates [15].

Parents’ characteristics also play a crucial role in the risk of child injuries. Children in socioeconomically disadvantaged families have higher rates of injuries. The risk factors include low family income, low parental education, single parenting, maternal age, older siblings, and area of residence [16,17,18,19,20,21,22,23]. Moreover, children whose parents were recurrent drinkers or had mental illness were at a higher risk of injury than other children [24]. Although past studies have identified the predictors related to child injuries with developmental disorders, national-level research in Asia is limited. The purpose of this research was to explore the risk factors for child injuries with developmental disorders in Taiwan using a national-level integrated database. On the basis of the identification of risk factors, intervention policies that aim to prevent child injuries can be further developed.

## 2. Materials and Methods

### 2.1. Study Design and Setting

This retrospective 1:1 nested case–control study was conducted using the linked data from the Maternal and Child Health Database (MCHD) for the years 2004–2014, National Health Insurance Research Database (NHIRD) for the years between 2004 and 2015, Census Registry for the years 2004–2014, and Indigenous Household Registration for the years 2006–2013. Children younger than 12 years old (born between 2004 and 2015) were enrolled in this study. These datasets are administrated by Health and Welfare Data Science Center, Ministry of Health and Welfare.

To identify emergency visits or hospitalizations due to injury, the ICD-9 diagnosis codes 800–904, 910–957, and 959–995, obtained from Ambulatory Care Expenditures and Inpatient Expenditures of the NHIRD, were used. The data on children with developmental disorders and substance use mental disorders among parents were collected from diagnoses in the NHIRD. In Taiwan, the government provides children aged 7 years or younger with health examinations, for 7 separate occasions. In addition, children aged 7 years or younger are provided with a variety of vaccines, free of charge. Children that are vaccinated, expected to make up a high portion of the entire population, are concurrently provided with physical and developmental evaluations [25]. The Census Registry provides the data on age and area of residence, while the data on Taiwanese indigenous peoples were confirmed by the Indigenous Household Registration. According to the Nationality Act, if one of the parents is a Taiwanese indigenous person, they can be recognized as an Indigenous. The data on family income were categorized according to parents’ registry for beneficiaries in the NHIRD.

**Figure 1:**
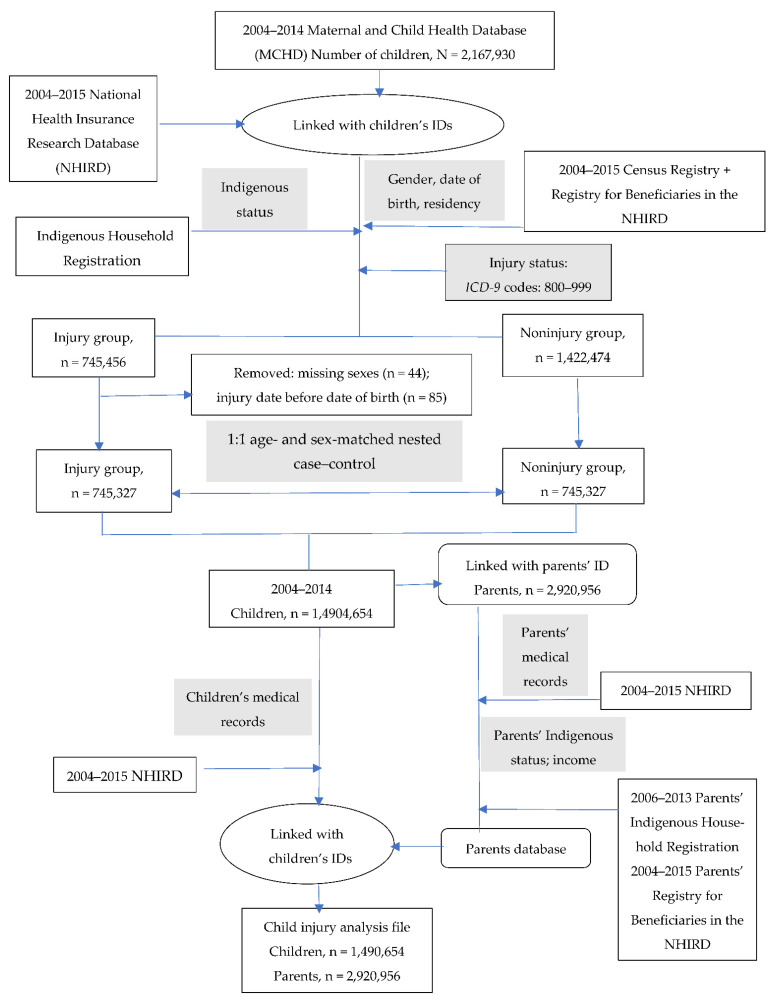
Study data flowchart.

Figure 1 illustrates the study flowchart. A total of 2,167,930 children between 2004–2014 were enrolled from the MCHD. Using children’s ID numbers, the MCHD and NHIRD were linked, yielding a total of 745,456 children who had records of visiting the ER or being hospitalized due to injury and 1,422,474 children without injury. We excluded cases with missing data. 

A 1:1 nested case–control study was performed, with 745,327 cases (injuries) and controls (noninjuries) obtained through age and sex matching. Finally, we obtained the child injury file, which contained 1,490,654 children and 2,920,956 parents.

This study was approved by the Taipei Medical University Joint Institutional Review Board (N201612015).

### 2.2. Variable Definitions

Developmental disorder was defined as the presence of any of the following: attention-deficit/hyperactivity disorder (ADHD), epilepsy, autism spectrum disorder, Tourette’s syndrome, and other developmental delay disorder. The related mental illnesses included temporary organic psychosis, schizophrenia, bipolar affective disorder, delusional state, other nonorganic psychoses, psychosis, anxiety state, hysteria symptoms, phobias, obsessive–compulsive disorder, psychiatric depression, neurasthenia, hypochondria, personality disorders, sexual psychopathy and disorders, psychogenic physical dysfunction, acute stress response, environmental adjustment disorder, special nonpsychotic mental illness after organic brain injury, melancholic disorder, and physical illness combined with psychological factors.

Area of residence was classified into 4 levels: highly urbanized area, moderately urbanized area, boomtown, and rural area. The township classification has been widely adopted in Taiwan [26].

Parents’ income was classified as low, middle, or high. The status of parents with alcohol and drug abuse was defined for those who were ever diagnosed and recorded in the NHIRD.

### 2.3. Statistical Analysis

We used the chi-square test to compare the proportion of factors between the injury and noninjury groups. A multiple logistic regression model was used to explore the associations between potential risk factors and child injury and to calculate the adjusted odds ratios (AORs). An alpha value of 0.05 was used, yielding a confidence level of 95%. A complete case analysis was performed in this study. Missing data were considered to be missing at random, and cases with missing data were excluded from the analysis.

## 3. Results

Table 1 presents the distribution of the injury/noninjury groups across risk factors. Several risk factors were more prevalent in the injury group than in the noninjury group: indigenous status (4.2% vs. 3.1%), developmental disorders in children (10.2% vs. 7.2%), parents with excessive alcohol use (0.8% vs. 0.6%), parents with illicit drug use (1.1% vs. 0.8%), parents with mental disorders (13.3% vs. 10.6%), rural residence (28.3% vs. 26.3%), and low parental income (22.8% vs. 21.8%).

Table 2 presents the results of logistic regression analysis for risk factors for child injuries. Children with developmental disorders were 49% more likely to have injuries than those without diseases (adjusted odds ratio [AOR]: 1.49, CI: 1.47–1.51). Indigenous children were at 29% higher risk of injury than non-indigenous children (AOR: 1.29, CI: 1.26–1.32). Those whose parents had illicit drug use and alcohol abuse had approximately 1.24 times (AOR: 1.24, CI: 1.16–1.32) and 1.11 times (AOR: 1.11, CI: 1.03–1.20) higher risk of injury, respectively. Children were 10%, 6%, and 26% more likely to sustain injuries if they were living in rural areas (AOR: 1.10; CI: 1.07–1.09) or if their parents were in the low-income group (AOR: 1.06; CI: 1.05–1.07) and had history of mental illness (AOR: 1.26; CI: 1.25–1.28).

We also performed subgroup analysis between children who visited ER/clinics or were hospitalized once and those with >1 visit/hospitalization (Table 3). Children with >1 visit had higher risks for injuries than those with only 1 visit in the following groups: indigenous status (AOR: 1.51 vs. AOR: 1.19), developmental disorders in children (AOR: 1.74; CI: 1.70–1.78), parents with illicit drug use (AOR: 1.48; CI: 1.32–1.66), parents with alcohol abuse (AOR: 1.21; CI: 1.07–1.37), and parents with history of mental illness (AOR: 1.43; CI: 1.41–1.46).

## 4. Discussion

To identify risk factors for injuries among children with development disorder, we conducted a 1:1 nested case-control study using the linked data from the MCHD, NHIRD, Census Registry, and Indigenous Household Registration. Several findings merit further discussion.

First, among children who have experienced hospital admission or ER/clinics visit due to injuries, the risk factors for child injury include the following: developmental disorders in children; indigenous status; residence in a rural area; and parents with illicit drug use, alcohol abuse, low income, and a history of mental illness. Based on the AORs, the top four major risk factors were developmental disorders in children, indigenous origin, parents with illicit drug use and alcohol abuse, and parents who were ever diagnosed with mental illness.

Second, the risk factors for children with repeated hospital admissions or ER/clinical visits due to injuries include: (1) developmental disorders in children; (2) indigenous origin; (3) parents with illicit drug use and alcohol abuse; and (4) parents ever having had mental illness. These were also the top four major risk factors for child injuries.

Our finding that children with developmental disorders were more injury-prone is in line with previous research in other countries. Children are particularly vulnerable to injuries as their activities in their surroundings increase before they develop the skills required to identify and respond to potential risks [27]. The reasons why children with disabilities have a higher risk of injury include deficiencies in motor skills, impaired causal reasoning, impaired mental processing, physical limitations, behavioral or emotional impairments, compromised adaptability, and potential adverse effects of medication used to treat their condition [8,28,29,30,31,32,33].

Indigenous children are at a high risk of injury. Pan et al. indicated that Taiwanese indigenous communities exhibit significantly higher risks of unintentional injury and death [34]. Such an effect has been observed in other countries [35]. The reasons for these findings are complex. The major risk factors for increased injury risks among indigenous children are cultural alienation and dispossession [36], low socioeconomic status [37,38,39], and geographical remoteness [37,40,41,42]. The socioeconomic status and educational level of the mother explained some of the differences between indigenous and nonindigenous children [43,44]. For indigenous peoples, several government policies or programs have been implemented to improve the environment for children. For instance, the Council of Indigenous Peoples had conducted the ‘Healthy and Safe Tribe’ program which was set up by local indigenous community-based organizations (CBOs) and their partners to prevent injuries and promote health within indigenous communities.

The detrimental effects of parental alcohol and drug abuse on children’s lives and injuries have been well documented. Parents with illicit drug use and alcohol abuse have a significant impact on child injuries, consistent with previous studies. Substance use can negatively affect the ways in which parents interact with and care for their children. Parents with substance use disorders may have the following characteristics, causing them to maltreat their children [45,46]: difficulties with regulating their own emotions and attending to their children’s emotions; diminished knowledge of parenting and child development; preoccupation with drug seeking; problems regulating their aggression; poor discipline skills (e.g., using coercive and harsh discipline); low levels of parental involvement; and lack of monitoring of children. 

Parental mental illness is also associated with an increased risk of child injury. Our result corroborates previous evidence [47] of a 14% increase in the risk of emergency hospital admissions and a 55% increase because of injuries among children living with adults with mental illness [48]. Mental illness in families is associated with relationship conflict and can lead to disruption of routines, unpredictable parenting, and inconsistent care [49]. Parents with a mental illness are less able to be responsive to the child and their environment [47,50] and may find it more challenging to be alert and maintain parental supervision.

Although some studies have indicated that socioeconomic factors influence the risk of child injury, our data indicate that lower family income and parental age have a marginal impact on the risks of child injury. This may be because of the existence of universal education in Taiwan, which offers to parents basic knowledge of child injury protection. Our conjecture here must be ascertained in future studies. In addition, in Taiwan, the Children’s Rights Law guarantees the basic safety of every child. Under the law, governments are required to set up a cross-sectoral coordinating mechanism to provide healthy and safe environments for children. These measures include, for example, children aged 6 years or below not being left alone at home, and traffic safety infrastructures being reinforced in the vicinity of schools. In addition, services such as counselling and investigation by government social workers are available for children with intentional injuries. Children’s basic right to subsistence will not differ due to differences in their family’s social and economic status.

Living in rural areas was also a risk factor for child injury. The result was similar to previous systematic review studies in the United States [51]. Such an effect may be because environmental conditions in rural areas, with activities such as farming, mining, fishing, or forestry, may make child more susceptible to injury. Another explanation may be the higher rate of injury from motor vehicle crashes among children living in rural areas [52].

The risks of unintentional injuries among children are mainly defined by individual factors (behaviors and attributes), the status of supervision, and safety environment and vehicle safety [53]. Intervention strategies to further prevent child injuries should be developed. For example, when a child is first diagnosed with a developmental disorder, health-care providers must be aware of the associated risk of injury and explain child injury prevention measures to the parents or caregivers. Policymakers should implement projects that make a community safer and enhance the awareness of family and child safety protection to reduce the incidence and severity of child injuries, especially in the indigenous communities. Reducing the symptoms related to drug and alcohol abuse and mental disorders is crucial in decreasing the risks of child injuries. For drug-addicted parents in particular, the Institute of Substance Treatment and Research in Taipei (ISTART) established in Taipei City sets up an integrated drug addiction medical center that develops a referral and triage system. The system aims to provide comprehensive treatments as early as possible.

Compared with past studies employing questionnaire interviews or analyzing small-scale data, the strength of this research is that a merged dataset at the national level was used, leading to data universality and reduced data collection bias. However, this study is not without its limitations. Data such as the course and severity of parents’ alcohol and substance abuse and parents’ mental illness, which are not available in our merged data, may potentially affect child injury risks. To obtain valuable insights into the underlying relationship between risk factors and child injuries among those with developmental disorders, additional subgroup analyses for particular risk factors, including rural areas, or parents with alcohol addiction or mental disorders, and their joint effects with disabled children on injuries, would be warranted.

## 5. Conclusions

In this study, the risk factors for child injury include the following: developmental disorders in children; indigenous status; residence in a rural area; and parents with illicit drug use, alcohol abuse, low income, and a history of mental illness. Furthermore, the major risk factors for repeated hospital admissions or ER/clinical visits include: having a developmental disorder; being indigenous; and having parents with illicit drug use, alcohol abuse, or history of mental illness. Health policies may include that healthcare providers should be aware of the risk factors identified in this study, and accordingly promote child injury prevention measures to parents or caregivers. Furthermore, reducing parents’ symptoms related to drug and alcohol abuse and mental disorders can be crucial in decreasing the risks of child injuries.

## Figures and Tables

**Table 1:** Distribution of child injury across a set of risk factors.

	Noninjury Group		Injury Group		*p* Value
	*n*	%	*n*	%	
Variable for children					
Indigenous status					<0.0001
No	721,874	96.9	713,749	95.8	
Yes	23,453	3.1	31,578	4.2	
Area of residence					<0.0001
Highly urbanized	148,507	21.1	142,424	20.3	
Moderately urbanized	209,572	29.8	208,726	29.7	
Boomtown ^a^	160,377	22.8	153,078	21.8	
Rural	184,281	26.3	198,164	28.3	
Development disorder ^b^					<0.0001
No	693,243	93.0	669,608	89.8	
Yes	52,084	7.0	75,719	10.2	
Parental variable					
Age					<0.0001
0–17	1455	0.1	937	0.0	
18–34	833,157	54.1	821,309	56.6	
35+	616,082	45.8	629,450	43.4	
Income					<0.0001
Low	306,804	21.8	320,093	22.8	
Medium	694,803	49.5	696,968	49.7	
High	399,755	28.5	383,161	27.3	
Illicit drug use					<0.0001
No	1,449,716	99.2	1,442,928	98.9	
Yes	12,103	0.8	16,209	1.1	
Alcohol addiction					<0.0001
No	1,452,832	99.4	1,446,975	99.2	
Yes	8987	0.6	12,162	0.8	
Mental disorder ^c^					<0.0001
No	1,307,371	89.4	1,268,599	86.7	
Yes	154,448	10.6	190,538	13.3	

^a^ According to several criteria (e.g., population density (people/km^2^), population ratio of people with college or above educational levels, population ratio of older people over 65 years old, population ratio of agriculture workers, and the number of physicians per 100,000 people), the townships in Taiwan were classified into highly urbanized area, moderately urbanized area, boomtown, and rural area. The most prosperous category, i.e., highly urbanized area, has the highest population density, the highest ratio of people with college or above educational levels, and the highest number of physicians per 100,000 people, whilst the least prosperous category, i.e., rural area, has the highest population ratio of older people over 65 years old and population ratio of agriculture workers. ^b^ developmental disorders include attention-deficit/hyperactivity disorder (ADHD), epilepsy, autism spectrum disorder, Tourette’s syndrome, and other developmental delay disorders. ^c^ Mental illness includes temporary organic psychosis, schizophrenia, bipolar affective disorder, delusional state, other nonorganic psychoses, psychosis, anxiety state, hysteria symptoms, phobias, obsessive–compulsive disorder, psychiatric depression, neurasthenia, hypochondria, personality disorders, sexual psychopathy and disorders, psychogenic physical dysfunction, acute stress response, environmental adjustment disorder, special nonpsychotic mental illness after organic brain injury, melancholic disorder, or physical illness combined with psychological factors.

**Table 2:** Results of logistic regression of risk factors for child injuries.

	AOR	95% CI
Variable for children		
Indigenous status		
No	ref.	
Yes	1.29 *	1.26–1.32
Area of residence		
Highly urbanized area	ref.	
Moderately urbanized area	1.04 *	1.03–1.05
Boomtown ^a^	0.99	0.98–1.00
Rural area	1.10 *	1.08–1.11
Development disorder ^b^		
No	ref.	
Yes	1.49 *	1.47–1.51
Parental variable		
Age		
0–17	0.67 *	0.58–0.78
18–34	ref.	
35+	1.06 *	1.05–1.08
Income		
Low	1.06 *	1.05–1.07
Medium	ref.	
High	0.93 *	0.92–0.94
Illicit drug use		
No	ref.	
Yes	1.24 *	1.16–1.32
Alcohol addiction		
No	ref.	
Yes	1.11 *	1.03–1.20
Mental disorder ^c^		
No	ref.	
Yes	1.26 *	1.25–1.28

^a^ According to several criteria (e.g., population density (people/km2), population ratio of people with college or above educational levels, population ratio of elder people over 65 years old, population ratio of agriculture workers, and the number of physicians per 100,000 people), the townships in Taiwan were classified into highly urbanized area, moderately urbanized area, boomtown, and rural area. The most prosperous category, i.e., highly urbanized area, has the highest population density, the highest ratio of people with college or above educational levels, and the highest number of physicians per 100,000 people, whilst the least prosperous category, i.e., rural area, has the highest population ratio of elder people over 65 years old and population ratio of agriculture workers. ^b^ developmental disorders include attention-deficit/hyperactivity disorder (ADHD), epilepsy, autism spectrum disorder, Tourette syndrome, and other developmental delay disorder. ^c^ mental illness includes temporary organic psychosis, schizophrenia, bipolar affective disorder, delusional state, other nonorganic psychoses, psychosis, anxiety state, hysteria Symptoms, phobias, obsessive–compulsive disorder, psychiatric depression, neurasthenia, hypochondria, personality disorders, sexual psychopathy and disorders, psychogenic physical dysfunction, acute stress response, environmental adjustment disorder, special nonpsychotic mental illness after organic brain injury, melancholic disorder, or physical illness combined with psychological factors. * *p* < 0.05.

**Table 3:** Results of logistic regression of risk factors for children who had ER/clinic visit or hospital admission (once vs. more than once).

	Once (*n* = 988,774)		More than Once (*n* = 501,880)	
	AOR	95% CI	AOR	95% CI
Variable for children				
Indigenous status				
No	ref.		ref.	
Yes	1.19 *	1.16–1.22	1.51 *	1.45–1.57
Area of residence				
Highly urbanized area	ref.		ref.	
Moderately urbanized area	1.04 *	1.02–1.05	1.04 *	1.02–1.06
Boomtown ^a^	1.00	0.99–1.02	0.96 *	0.94–0.98
Rural area	1.08 *	1.06–1.10	1.14 *	1.11–1.17
Development disorder ^b^				
No	ref.		ref.	
Yes	1.34 *	1.32–1.37	1.74 *	1.70–1.78
Parental variable				
Age				
0–17	0.61 *	0.50–0.75	0.73 *	0.58–0.91
18–34	ref.		ref.	
35+	1.08 *	1.06–1.09	1.04 *	1.02–1.06
Income				
Low	1.05 *	1.03–1.06	1.09 *	1.06–1.11
Medium	ref.		ref.	
High	0.93 *	0.91–0.94	0.95 *	0.93–0.97
Illicit drug use				
No	ref.		ref.	
Yes	1.12 *	1.03–1.22	1.48 *	1.32–1.66
Alcohol addiction				
No	ref.		ref.	
Yes	1.06	0.97–1.17	1.21 *	1.06–1.37
Mental disorder ^c^				
No	ref.		ref.	
Yes	1.18 *	1.17–1.20	1.43 *	1.41–1.46

^a^ According to several criteria (e.g., population density (people/km^2^), population ratio of people with college or above educational levels, population ratio of elder people over 65 years old, population ratio of agriculture workers, and the number of physicians per 100,000 people), the townships in Taiwan were classified into highly urbanized area, moderately urbanized area, boomtown, and rural area. The most prosperous category, i.e., highly urbanized area, has the highest population density, the highest ratio of people with college or above educational levels, and the highest number of physicians per 100,000 people, whilst the least prosperous category, i.e., rural area, has the highest population ratio of elder people over 65 years old and population ratio of agriculture workers. ^b^ developmental disorders include attention-deficit/hyperactivity disorder (ADHD), epilepsy, autism spectrum disorder, Tourette syndrome, and other developmental delay disorder. ^c^ mental illness includes temporary organic psychosis, schizophrenia, bipolar affective disorder, delusional state, other nonorganic psychoses, psychosis, anxiety state, hysteria Symptoms, phobias, obsessive–compulsive disorder, psychiatric depression, neurasthenia, hypochondria, personality disorders, sexual psychopathy and disorders, psychogenic physical dysfunction, acute stress response, environmental adjustment disorder, special nonpsychotic mental illness after organic brain injury, melancholic disorder, or physical illness combined with psychological factors. * *p* < 0.05.

## Data Availability

The data that support the findings of this study are available from the Health and Welfare Data Science Center of the Ministry of Health and Welfare but restrictions apply to the availability of these data, which were used under license for the current study, and so are not publicly available.
